# Biologic-free remission by orthopaedic surgery in non-responder to infliximab for rheumatoid arthritis

**DOI:** 10.1186/s40064-015-1397-5

**Published:** 2015-10-13

**Authors:** Katsuaki Kanbe, Junji Chiba, Yasuo Inoue, Masashi Taguchi, Akiko Yabuki

**Affiliations:** Department of Orthopaedic Surgery, Tokyo Women’s Medical University, Medical Center East, 2-1-10 Nishiogu, Arakawa, Tokyo, 116-8567 Japan

**Keywords:** Infliximab, Biologic-free, Remission, Orthopaedic surgery, Rheumatoid arthritis

## Abstract

The aim of this study was to investigate remission and biologic-free remission after orthopaedic surgery and related clinical factors in non-responder to infliximab for rheumatoid arthritis (RA). We analyzed 74 patients who were treated with 3 mg/kg infliximab and methotrexate and underwent orthopaedic surgery after non-responder to infliximab with disease activity score (DAS) 28 (CRP) of ≥3.2. The rates of remission and biologic-free remission at 52 weeks after orthopaedic surgery were investigated and the clinical factors related to remission and biologic-free remission were analyzed by logistic regression and receiver-operating characteristic analyses. The rates of total remission and biologic-free remission were 37/74 (50 %) and 9/74 (12.2 %), respectively. Regarding orthopaedic surgery, the rates of remission and biologic-free remission were 25/38 (65.8 %) and 7/38 (18.4 %) for synovectomy, 7/20 (35 %) and 0/20 (0 %) for arthroplasty, and 5/16 (31.3 %) and 2/16 12.5) for others including spine surgery and foot surgery. DAS28(CRP) at baseline was significantly related to both remission and biologic-free remission. Prednisolone was negatively associated with remission, and DAS28(CRP) was related to biologic-free remission by logistic regression analyses. DAS28(CRP) below 3.7 was cutoff point for acquiring biologic-free remission of non-responder to infliximab after orthopaedic surgery. Therefore orthopaedic surgery may be effective to obtain remission or biologic-free remission in RA patients treated with biologics.

## Background

Anti-tumor necrosis factor (TNF)-α therapy such as infliximab for rheumatoid arthritis (RA) is used not only to inhibit inflammation, but also to suppress bone and joint destruction. It is reported that infliximab leads biologic-free remission in the long term for early RA patients with good responses (van der Kooij et al. 2009; Bejarano et al. 2010; Quinn et al. 2005; van der Bijl et al. 2007; van den Broek et al. 2011). However, it is difficult to induce biologic-free remission in non-responder to infliximab. No reports to date have described how orthopaedic surgery contributes to remission or biologic-free remission. Anti-TNF-α therapy cannot improve the clinical outcomes in some patients with joint swelling and tenderness with bone destruction and synovium proliferation, and such patients are indicated to undergo orthopaedic surgery, such as total arthroplasty or arthroscopic synovectomy (Momohara et al. 2011; Kanbe and Inoue 2006). However, it remains unclear which orthopaedic surgeries are suitable for the induction of remission as well as biologic-free remission in infliximab treatment, and how clinical factors are related to these outcomes. In this study, we treated 74 patients by orthopaedic surgery among cases with non-responder to infliximab [disease activity score (DAS) 28 (CRP) of ≥3.2]. Retrospective and case studies were analyzed to detect specific factors related to remission and biologic-free remission after orthopaedic surgery under treatment with infliximab in RA. This is the first report related to bio-free remission by orthopaedic surgery for RA.

## Methods

### Patients and methods

Seventy-four patients (9 males and 65 females) with a mean age of 60.4 ± 1.05 years, mean disease duration of 12.29 ± 11.78 years, mean disease activity score (DAS) 28 (CRP) of 4.32 ± 0.71, mean CRP of 2.67 ± 3.22 mg/dL, mean methotrexate (MTX) dose of 6.02 ± 1.17 mg/week, and mean prednisolone (PSL) dose of 3.68 ± 2.29 mg/day were treated by orthopaedic surgery in non-responder to 3 mg/kg infliximab for a mean period of 1.15 ± 0.53 years (Table 1). The 74 orthopaedic surgeries included 38 synovectomy procedures in 33 knees, three shoulders, one elbow, and one wrist, 20 arthroplasty procedures comprising 8 total knee arthroplasties (TKA), three total hip arthroplasties (THA), five total shoulder arthroplasties (TSA), three total elbow arthroplasties (TEA), and one total ankle arthroplasty (TAA), and 16 other procedures comprising six spine surgeries, eight foot surgeries, and two hand surgeries. The rehabilitation was also performed after all orthopaedic procedures. The treatment of infliximab was stopped around 4 weeks before surgery and restarted approximately around 4 weeks after surgery. MTX was stopped only one week of surgery, however PSL was continued. The infliximab treatment included a diagnosis of RA based on the American College of Rheumatology (ACR) criteria (Arnett et al. 1988), and categorization according to Steinbrocker et al. (Steinbrocker et al. 1949). The disease stages were stage II in 16 patients, stage III in 40 patients, and stage IV in 18 patients. The classes were class 2 in 20 patients, class 3 in 46 patients, and class 4 in 8 patients. At 52 weeks after surgery, the DAS28(CRP) was analyzed for the rates of remission, DAS28(CRP) of ≤2.6, and biologic-free remission (Hirata et al. 2013; van der Heijde et al. 1990). Biologic-free remission was obtained after remission accomplished at 24 weeks after surgery. Logistic regression analyses were performed for age, disease duration, DAS28(CRP), dose of PSL, dose of MTX, stages and class at baseline. Receiver-operating characteristic (ROC) analyses were performed to acquire the cutoff points with sensitivity and specificity related to the significant clinical factors. Informed consent was obtained from all patients, and the study protocol was approved by the ethics committee of Tokyo Women’s Medical University (approval number 1321).

**Table 1:** Patients backgrounds at base line for orthopaedic surgery

N	74
Age (years)	60.4 ± 1.05
Female (%)	65/74 (87.8 %)
DD (years)	12.29 ± 11.78
DAS28(CRP)	4.32 ± 0.71
CRP (mg/dl)	2.67 ± 3.22
MTX (mg/week)	6.02 ± 1.17
PSL (mg/day)	3.68 ± 2.29
Stage/class	II; 16, III; 40, IV; 18/2; 20, 3; 46, 4; 8
Infliximab (years)	1.15 ± 0.53
Orthopaedic surgery	Synovectomy 38
	Arthroplasty 20
	Others 16

*DD* disease duration, *DAS* disease activity score, *CRP* c-reactive protein, *MTX* methotrexate, *PSL* predonisolone

### Statistical analysis

The Wilcoxon signed-rank test was used to compare the DAS28(CRP) scores at baseline and 52 weeks after orthopaedic surgery. Logistic regression analyses related to remission and biologic-free remission were, respectively carried out at 52 weeks using StatFlex version 6.0 (Statflex, Tokyo, Japan). ROC curves were calculated to acquire the sensitivity and specificity with odds ratios of the clinical factors using the above software. Values of *p* < 0.05 were considered significant.

## Results

**Fig. 1:**
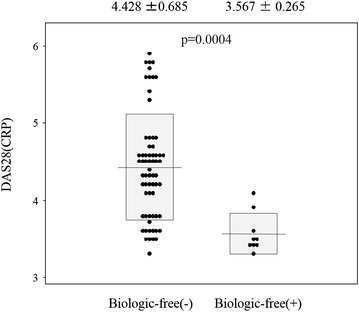
DAS28(CRP) values after orthopaedic surgery in non-responder to the cases of infliximab at 52 weeks regarding biologic-free remission

**Fig. 2:**
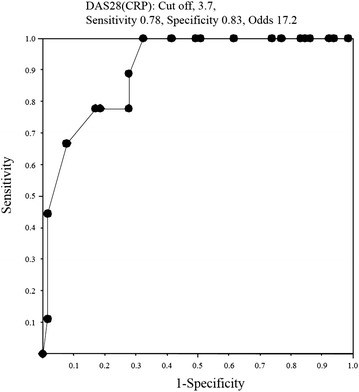
ROC curves of biologic-free remission after orthopaedic surgery to analyze the cutoff points, sensitivities, specificities, and odds ratios regarding to DAS28(CRP)

The remission rate of the total patients at 52 weeks was 37/74 (50 %), including 25/38 (65.8 %) for synovectomy, 7/20 (35 %) for arthroplasty, and 5/16 (31.3 %) for others (Table 2). The biologic-free remission rate was 9/74 (12.2 %), including 7/38 (18.4 %) for synovectomy, 0/20 (0 %) for arthroplasty, and 2/16 (12.5 %) for others (Table 2). The Boolean remission was 27/74 (36.5 %) and bio-free Boolean remission was 8/74 (10.8 %) after surgery. The rates of remission and biologic-free remission continued, being 19/37 (51.4 %) and 7/9 (77.8 %) at 104 weeks after orthopaedic surgery, respectively. The logistic regression analyses showed that DAS28(CRP) at baseline (*p* = 0.0025) and PSL (*p* = 0.0348) were related to remission (Table 3). Therefore, low DAS28(CRP) at baseline and low dose of PSL were significantly correlated with remission. However, the logistic regression analyses showed that DAS28(CRP) at baseline (*p* = 0.0401) were related to biologic-free remission (Table 4). Therefore, low DAS28(CRP) at baseline was significantly correlated with biologic-free remission after orthopaedic surgery. The DAS28(CRP) at baseline differed significantly, being 4.428 ± 0.685 in the no biologic-free remission group and 3.567 ± 0.265 in the biologic-free remission group (*p* < 0.0004) (Fig. 1). The ROC analyses showed that the cutoff point for DAS28(CRP) was 3.7, with sensitivity of 0.78, specificity of 0.83, and odds ratio of 17.2 (Fig. 2). Therefore, DAS28(CRP) below 3.7 was significantly correlated with obtaining biologic-free remission after orthopaedic surgery in treatment of non-responder to infliximab for RA (Fig. 2).

**Table 2:** Remission and bio-free rate after orthopaedic surgery

	Total	Synovectomy	Arthroplasty	Others
Remission rate	37/74 (50 %)	25/38 (65.8 %)	7/20 (35 %)	5/16 (31.3 %)
Bio-free rate	9/74 (12.2 %)	7/38 (18.4 %)	0/20 (0 %)	2/16 (12.5 %)

**Table 3:** Clinical factors related to the remission

Factors	P	Odds	95 % CI
Age	0.5574	0.97981	0.91525–1.04892
DD	0.7197	0.99877	0.99207–1.00551
DAS28	0.0025	0.03237	0.00351–0.29830
PSL	0.0348	0.53672	0.30112–0.95665
MTX	0.6036	1.26905	0.51632–3.11920
Stage	0.6344	1.56574	0.24664–9.93964
Class	0.1442	0.33875	0.07925–1.44801

*DD* disease duration, *DAS* disease activity score, *PSL* predonisolone, *MTX* methotrexate

**Table 4:** Clinical factors related to the biologic-free remission

Factors	P	Odds	95 % CI
Age	0.0875	1.27742	0.96462–1.69165
DD	0.3805	0.9947	0.98294–1.00659
DAS28	0.0401	0.00284	0.00001–0.76627
PSL	0.3289	1.53217	0.65064–3.60805
MTX	0.9742	0.98265	0.34078–2.83353
Stage	0.8398	0.64611	0.00936–44.5802
Class	0.9247	1.18464	0.03524–39.8251

*DD* disease duration, *DAS* disease activity score, *PSL* predonisolone, *MTX* methotrexate

## Discussion

Biologic-free treatment in RA was first reported in the TNF20 study (Bejarano et al. 2010; Quinn et al. 2005). Patients with early RA who had experienced symptoms for <12 months were treated with a combination of infliximab and MTX. Patients who initiated treatment with infliximab and MTX achieved higher American College of Rheumatology 50 and 70 % improvement responses than patients who initiated therapy with MTX and placebo. At 1 year after stopping the induction therapy, the response was sustained in 70 % of patients who received infliximab and MTX. In the Best study, it was described that infliximab had potential for biologic-free remission in the long-term results, with 56 % discontinuation of infliximab at ≥6 months and 52 % at 7.2 years (van der Kooij et al. 2009; van der Bijl et al. 2007; van den Broek et al. 2011). Other biologics such as adalimumab were also reported for biologic-free remission, as 58 % of patients achieved adalimumab-free remission at the primary end point of 6 months after discontinuation of adalimumab (Felson et al. 2011). However, there is no evidence for acquisition of remission or biologic-free remission of non-responder to infliximab in RA. We previously reported that synovectomy was effective for non-responder cases of infliximab to improve the DAS28 after surgery (Kanbe and Inoue 2006). Arthroplasty was also reported as a method for restoring biologic efficacy (Momohara et al. 2011). Although the mechanism for non-responder to infliximab is unknown, serum levels of IL-6 were upregulated, even with the use of TNF-α blockers (Takeuchi et al. 2012). It was reported that TNF-α expression in the synovium was correlated with DAS28(CRP) by immunohistochemistry (Kanbe et al. 2013). Orthopaedic surgery may have a role in removing the synovium containing TNF-α and other cytokines, thereby reducing the inflammation to maintain biologic efficacy. This study showed 50 % remission after orthopaedic surgery even under non-responder of infliximab and 12.2 % biologic-free remission. In particular, synovectomy was more effective than other surgical treatments, meaning that direct removal of synovial tissue might be useful for infliximab toleration. The baseline DAS28(CRP) played a role in predicting the efficacy of orthopaedic surgery for remission as well as biologic-free remission, even for DAS28(CRP) of ≥3.2. Thus, lower disease activity in non-responder to infliximab was suitable for application of surgery to acquire biologic-free remission. Furthermore, early timing of orthopaedic surgery after toleration of infliximab may be important to obtain a high rate of remission before increasing DAS28(CRP). The rates of remission and biologic-free remission continued at 51.4 and 77.8 %, respectively, at 104 weeks after orthopaedic surgery. Thus, biologic-free remission was stable for a relatively long period in this study. In the ROC analyses, we found that DAS28(CRP) below 3.7 showed high potential for obtaining biologic-free remission in non-responder to infliximab after orthopaedic surgery. This indicated that low disease activity may require restoration of joint functions by orthopaedic surgery before withdrawing biologics.

The limitations of this study are related to its retrospective nature, including the small number of patients (*n* = 74) and absence of a non-operative control group for comparison with cases of DAS28(CRP) of ≥3.2. Future studies should consider the long-term results of biologic-free remission cases after orthopaedic surgery, and joint destruction by X-ray assessment should be evaluated.

## Conclusions

Orthopaedic surgery was effective for non-responder to infliximab treated cases to obtain remission or biologic-free remission among RA patients with low disease activity.
